# Case Report: Radioactive Holmium-166 Microspheres for the Intratumoral Treatment of a Canine Pituitary Tumor

**DOI:** 10.3389/fvets.2021.748247

**Published:** 2021-11-04

**Authors:** Nino Chiron Morsink, Nienke Johanna Maria Klaassen, Björn Petrus Meij, Jolle Kirpensteijn, Guillaume Cornelis Maria Grinwis, Irene Afra Schaafsma, Jan Willem Hesselink, Johannes Frank Wilhelmus Nijsen, Sebastiaan Alexander van Nimwegen

**Affiliations:** ^1^Department of Clinical Sciences, Faculty of Veterinary Medicine, Utrecht University, Utrecht, Netherlands; ^2^Department of Medical Imaging, Radboud Institute for Health Sciences, Radboud University Medical Center, Nijmegen, Netherlands; ^3^Department of Biomolecular Health Sciences, Faculty of Veterinary Medicine, Utrecht University, Utrecht, Netherlands; ^4^Quirem Medical, Deventer, Netherlands

**Keywords:** brachytherapy, canine, dog, holmium, intratumoral treatment, pituitary, radionuclide, translational model

## Abstract

**Introduction:** In this case study, a client-owned dog with a large pituitary tumor was experimentally treated by intratumoral injection of radioactive holmium-166 microspheres (^166^HoMS), named ^166^Ho microbrachytherapy. To our knowledge, this is the first intracranial intratumoral treatment through needle injection of radioactive microspheres.

**Materials and Methods:** A 10-year-old Jack Russell Terrier was referred to the Clinic for Companion Animal Health (Faculty of Veterinary Medicine, Utrecht University, The Netherlands) with behavioral changes, restlessness, stiff gait, and compulsive circling. MRI and CT showed a pituitary tumor with basisphenoid bone invasion and marked mass effect. The tumor measured 8.8 cm^3^ with a pituitary height-to-brain area (P/B) ratio of 1.86 cm^−1^ [pituitary height (cm) ×10/brain area (cm^2^)]. To reduce tumor volume and neurological signs, ^166^HoMS were administered in the tumor center by transsphenoidal CT-guided needle injections.

**Results:** Two manual CT-guided injections were performed containing 0.6 ml of ^166^HoMS suspension in total. A total of 1097 MBq was delivered, resulting in a calculated average tumor dose of 1866 Gy. At 138 days after treatment, the tumor volume measured 5.3 cm^3^ with a P/B ratio of 1.41 cm^−1^, revealing a total tumor volume reduction of 40%. Debulking surgery was performed five months after ^166^HoMS treatment due to recurrent neurological signs. The patient was euthanized two weeks later at request of the owners. Histopathological analysis indicated a pituitary adenoma at time of treatment, with more malignant characteristics during debulking surgery.

**Conclusion:** The 40% tumor volume reduction without evident severe periprocedural side effects demonstrated the feasibility of intracranial intratumoral ^166^HoMS treatment in this single dog.

## Introduction

Neoplasms of the pituitary gland and craniopharyngeal duct accounted for 17.8% of 405.740 human brain and central nervous system (CNS) tumors in the United States between 2012 and 2016 (1). Pituitary adenomas may result in symptoms due to mass effect and abnormal hormone secretion, whereas pituitary carcinomas are characterized by metastasis and account for only 0.1–0.2% of pituitary tumors in humans (2). Current treatment options include medication, chemotherapy, surgery, and radiation therapy (RT) (3–6). Most pituitary neoplasms do not respond to medication alone and require multimodal therapy (7). Unfortunately, surgery often results in residual disease because of incomplete resection (8), and radiotherapy (RT) is often limited by potential radiation damage to healthy tissue (9, 10). As a result, the current treatment options are mainly palliative, with a 5-year overall survival rate of 79.0% for adenomas and 28.6% for carcinomas in humans (11–14).

Intratumoral injection of radioactive holmium-166 microspheres (^166^HoMS), ^166^Ho microbrachytherapy, is a new, minimally invasive treatment for solid malignancies which has been investigated in patients with no other effective treatment available (15–18). ^166^Ho is considered an ideal radioisotope for intratumoral treatment of solid tumors due to the high dose rate, multimodality imaging options, and relatively short half-life (t_1/2_ = 26.8 h) (19). ^166^Ho emits high-energy beta radiation (E_β, *max*_ = 1.85 MeV) with a relatively short penetration depth in tissue (average 2.2 mm, maximum 8.7 mm) (20, 21), which enables a high radiation dose in a single treatment with minimal radiation exposure to surrounding tissues (22). Intratumoral ^166^HoMS administration proved to be a safe and potentially effective treatment option in feline oral squamous cell carcinoma (SCC) and liver malignancies (15, 22), and in head and neck SCC in human patients (16), resulting in extremely high tumor-absorbed doses without evident treatment-related side effects.

In this case study, the first palliative intracranial ^166^Ho microbrachytherapy is described in a Jack Russell Terrier with a large pituitary tumor, with the aim to reduce tumor volume, to improve the dog's quality of life, and to evaluate the feasibility of this treatment.

## Case Description

A 10-year-old male neutered Jack Russell Terrier (8.9 kg) was referred to the Clinic for Companion Animal Health (Faculty of Veterinary Medicine, Utrecht University, The Netherlands) with behavioral changes, disorientation, restlessness, stiff gait, weight loss, and compulsive circling. The dog had separation anxiety and occasionally showed inappropriate urination. Neurological examination indicated that clinical signs originated from the CNS. MRI (Magnetom Open Viva 0.2T, Siemens Healthineers, Erlangen, Germany) revealed a mass originating from the pituitary fossa measuring 28.1 mm (height), 17.1 mm (width), and 24.0 mm (length), resulting in a calculated ellipsoid tumor volume of 6.0 cm^3^ according to Equation 1.


(1) $$\begin{array}{r} \text{Volume }=\frac{\pi }{6}\times \text{length}\times \text{width}\times \text{height} \end{array}$$

The mass had well-defined, mildly undulating margination and was strongly contrast-enhancing with marked mass effect on the adjacent hypothalamus, causing dorsal displacement of the third ventricle and mild distension of the third and lateral ventricles. An invasive nonfunctioning adenoma was considered most likely because of mid-sagittal bony lysis of the basisphenoid bone with minimal protrusion into the nasopharynx. There were no intracranial metastases detected; however, imaging was not performed to rule out a pituitary carcinoma based on possible extracranial metastases (23). Biochemical and hematological blood analysis showed no abnormalities. Prednisone treatment was started (Prednoral, AST Farma, Oudewater, The Netherlands) and continued on effect, resulting in significant clinical improvement.

**Figure 1:**
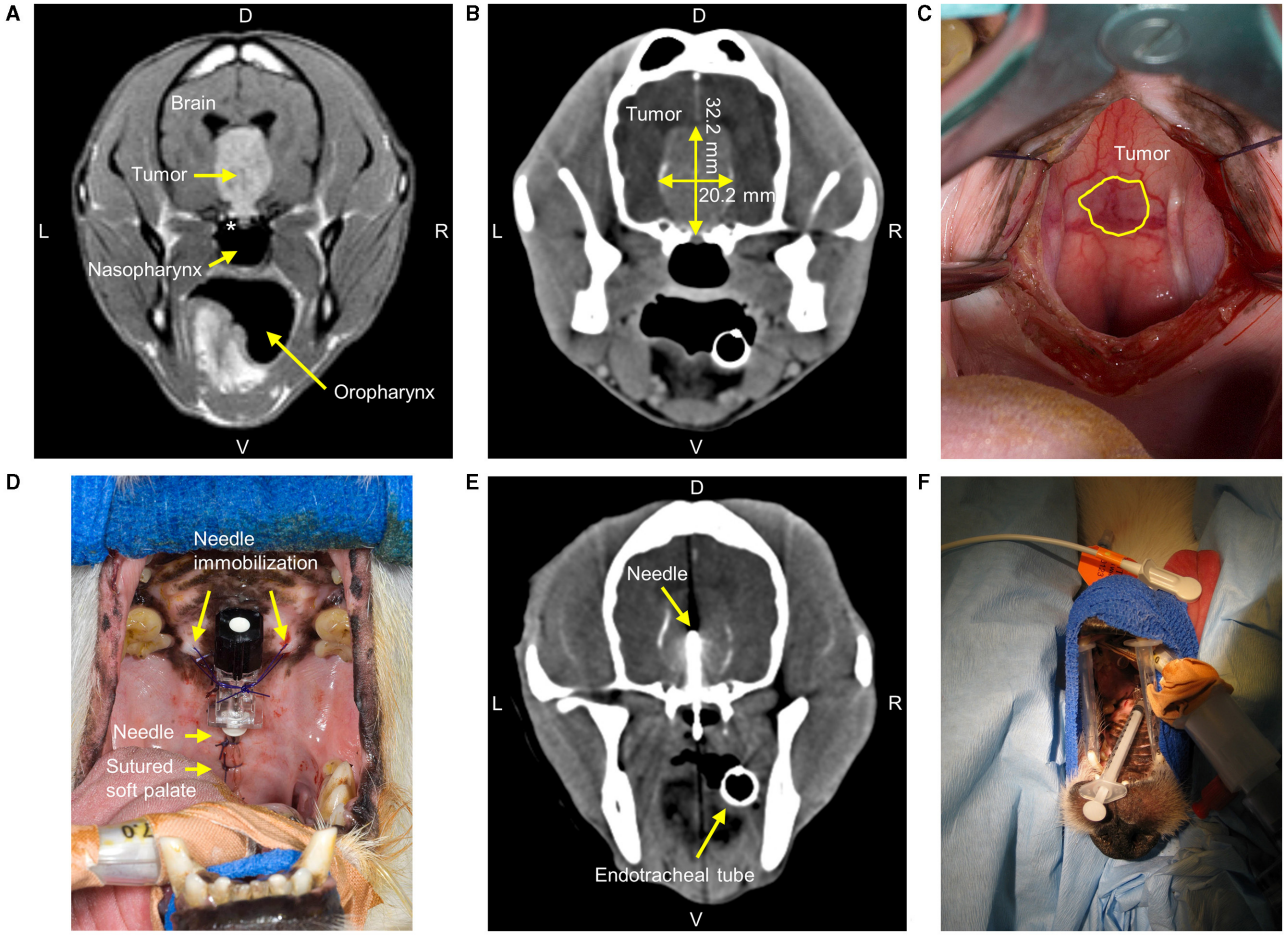
Diagnostic imaging and intratumoral holmium-166 microspheres treatment of a 10-year-old Jack Russell Terrier with a pituitary tumor. **(A)** Gadolinium-enhanced T1-weighted spin-echo MRI six months before treatment, showing a large hyperintense circumscribed pituitary mass lesion with basisphenoid bone invasion (*). L = Left, R = Right, D = Dorsal, V = Ventral. **(B)** Contrast-enhanced CT (2 mm interslice spacing with 0.5 mm overlap, tube current 200 mA, tube voltage 120 kV) immediately before treatment with longest tumor height and width measured. **(C)** During the transsphenoidal approach and after retraction of the soft palate, the tumor was visible through the mucoperiosteum. **(D)** The needle was placed into the tumor center and immobilized with sutures after closure of the soft palate. **(E)** CT of the skull shows the needle as an imaging artifact with the needle tip in the tumor center. **(F)** The syringe was attached to the prepositioned immobilized needle for ^166^HoMS administration in the tumor center. The dog was positioned on his back.

Sixteen months later, the dog returned because of minimal signs consistent with Cushing's syndrome, possibly iatrogenic due to prolonged steroid treatment. MRI (Figure 1A) showed no significant changes compared to the previous MRI and intermittent prednisone treatment was continued.

Twenty-two months after initial presentation, the dog suffered from recurring neurological signs including behavioral changes, restlessness, stiff gait, and compulsive circling. Additionally, the dog slept longer and deeper than usual, and frequently folded the ears backwards and squinted the eyes, possibly indicating headaches (24). Neurological examination revealed the absence of proprioception in the hind legs and prednisone dosage was increased (0.5 mg/kg once daily). Complete surgical resection was not considered possible and debulking surgery was not advised because of the anticipated high risk of complications. Based on our experience with ^166^HoMS treatment of other tumors and the transsphenoidal accessibility of the lesion, intratumoral needle injection of ^166^HoMS through a transsphenoidal approach was performed (15, 16, 18, 25).

## Timeline

A timeline of the episode of care is presented in Table 1.

**Table 1:** Timeline showcasing relevant data from episodes of care.

**Visits**	**Diagnostics**	**Findings**	**Treatment/Intervention**
Month −22 Presentation	- Initial consultation. - General and neurological exam. - Biochemical and hematological blood analysis. - MRI.	6.0 cm^3^ suspected pituitary adenoma with bony lysis and protrusion into the nasopharynx. No intracranial metastases.	Prednisone 0.5 mg/kg twice daily, to once daily, to once every other day.
Month −6 Follow-up	- General and neurological exam. - MRI.	Minimal clinical signs.Stable presentation on MRI.	Prednisone 0.5 mg/kg once every other day/on effect.
Day 0 Treatment	- General and neurological exam. - Biochemical and hematological blood analysis. - CT.	Worsening of clinical signs with 10.6 cm^3^ tumor on pretreatment CT.	CT-guided intratumoral needle injections of ^166^HoMS.
Day +24 Follow-up	- General and neurological exam. - Biochemical blood analysis.	Improved clinical signs, but lethargic and polyuric/polydipsic.	Phenobarbital discontinued.
Day +41 Follow-up	- General and neurological exam. - CT and MRI.	Return neurological signs despite decrease in tumor height/width.	Prednisone 1 mg/kg twice daily.
Day +138 Follow-up	- General and neurological exam. - Biochemical and hematological blood analysis. - CT.	Worsening of clinical signs.	Transsphenoidal hypophysectomy.
Day +154 Euthanasia		Initially improved neurological signs, followed by rapid decline.	Euthanasia at request of the owners by their own veterinarian.

*^166^HoMS = holmium-166 microspheres.*

## ^166^HoMS Treatment

### Anesthesia, Analgesia, Medication

General anesthesia was induced by IV administration of propofol (3 mg/kg; Propovet, AST Farma, Oudewater, The Netherlands) and maintained by inhalation of isoflurane (1.5-2.0%) in O_2_/air (1:1). The patient was continuously monitored (heart rate, respiratory rate and effort, temperature, pulse oximetry, blood pressure, capnography) and perioperative medication was administered according to the protocol for transsphenoidal hypophysectomy as previously described (26–28). Additional medication included the IV administration of cefovecin sodium (8 mg/kg; Convenia, Zoetis, Louvain-la-Neuve, Belgium), methylprednisolone (5 mg/kg; Solu-Medrol, Pfizer, New-York, USA), and phenobarbital (2 mg/kg twice daily; Veterinary Medicine Pharmacy, Utrecht, The Netherlands).

### Pretreatment CT

To assess tumor dimensions and identify anatomical landmarks for needle positioning, a preoperative contrast-enhanced CT (2 mm slices with 0.5 mm overlap, 200 mA, 120 kV; Secura, Philips, Eindhoven, The Netherlands) was performed following a pituitary imaging protocol (29). The CT (Figure 1B) showed intratumoral mineralization and the tumor now measured 32.2 mm (height), 20.2 mm (width), and 31.1 mm (length), resulting in 10.6 cm^3^ according to Equation 1, compared to 6.0 cm^3^ on MRI six months earlier.

### Holmium Microsphere and Syringe Preparation

We aimed for a tumor-absorbed dose of 800 Gy, as previously used in a high-dose patient cohort in the intratumoral treatment of feline oral SCC (15). Based on 10.6 cm^3^ tumor volume from the pretreatment CT, the minimum required amount of injected radioactivity was 566 MBq according to Equation 2. *A* = ^166^Ho radioactivity in MBq; *D* = tumor-absorbed dose in Gray (Gy [J/kg]); ^166^Ho-specific tissue dose conversion coefficient = 15.87 mJ/MBq; *W* = tumor weight (g). Assumed tumor tissue density was 1.06 g/cm^3^ (30, 31).


(2) $$\begin{array}{l} A=\frac{D\times W}{15.87} \end{array}$$

Holmium acetylacetonate microspheres (5-40 μm) were produced at the University Medical Center Utrecht (Utrecht, The Netherlands) in compliance with good manufacturing practice as previously described (19, 32). The HoMS were neutron irradiated at the Reactor Institute Delft (Delft University of Technology, The Netherlands) and transported to the clinic. Of the ^166^HoMS, 200 mg was suspended in 0.6 ml sterile water containing 2% poloxamer 188 (Pluronic F-68, Sigma-Aldrich Chemie, Zwijndrecht, The Netherlands) by repeatedly drawing the suspension up and down with a syringe. Two aliquots of 0.3 ml were drawn up into two 1-ml Luer-lock syringes. Based on previous intratumoral applications, we anticipated a 50% “loss” of ^166^HoMS during syringe preparation and treatment (15) and we aimed to prepare double the amount of the required activity (1132 MBq). Using a dose calibrator (VDC-404, Comecer, Joure, The Netherlands), the two prepared syringes measured 355 and 853 MBq (1208 MBq in total) at time of treatment. Each syringe was placed into an acrylic glass cylinder to limit beta radiation exposure of personnel during syringe preparation and ^166^HoMS administration.

### Transsphenoidal Needle Placement

During the transsphenoidal approach (26) and after electrosurgical incision of the soft palate, the tumor was readily visible through the mucoperiosteum (Figure 1C). The mucoperiosteum was coagulated and excised at the outer tumor boundary. Several small (2–3 mm) biopsies were taken from the ventral tumor part and fixed in 10% neutral-buffered formalin. A cylindrical ø8 × 6 mm hemostatic sponge (Spongostan, Ethicon, New Jersey, USA) and bone wax were placed to close the defect in the basisphenoid bone. A 22G × 40 mm needle (Spinocan, B. Braun, Melsungen, Germany) was bent ~90° and placed with its tip in the tumor center. The needle was immobilized using bone wax, several PDS 3-0 (Ethicon) sutures, and the soft palate sutured around the needle (Figure 1D). CT was repeated and showed correct needle placement with the tip in the tumor center (Figure 1E). The patient was brought to the radionuclide facility for ^166^HoMS administration.

### ^166^HoMS Administration

Because the ^166^HoMS sedimented in the syringes, the two prefilled syringes were gently shaken before administration to create a visually homogeneous suspension. The syringes were immediately attached to the prepositioned needle and the ^166^HoMS suspensions were manually injected in ~2 sec (Figure 1F). Gauze sponges were placed against the soft palate to collect potential leakage out of the injection canal. ^166^HoMS administration was performed without observed treatment complications. There was no observed leakage out of the needle tract, no needle blockage due to ^166^HoMS precipitation, and no evident injection resistance.

### Postoperative Care

After treatment, the patient was housed in the radionuclide ward and monitored daily by general and neurological assessment, and by biochemical and hematological blood analysis. Additional postoperative medication included IM administration of methylprednisolone (1 mg/kg; Moderin Long-Acting, Zoetis, Louvain-la-Neuve, Belgium) and phenobarbital treatment continued orally in a tapering dose schedule.

The dog was discharged after two and a half days, when the dose rate was below the regulatory limit of 1 μSv/h at 1 m, measured using a dose rate meter (RDS-100, Alnor, Minnesota, USA). The compulsive circling had steadily decreased during hospitalization. Upon discharge, the dog was eating and drinking, and had normal urination and defecation. Despite seeming lethargic, the dog appeared calmer and more alert. The owner received radiation safety instructions for the care of their dog in the first week after treatment. At home, general and neurological signs were monitored daily by the owners by qualitative assessment according to specific questions and instructions from the clinician. Follow-up was planned approximately three and six weeks after ^166^HoMS treatment, including diagnostic imaging after six weeks.

## Follow-Up

The dog returned to the clinic 24 days after ^166^HoMS treatment. Clinical signs that are considered related to headache (squinting of the eyes and folding of the ears) had decreased, whereas the compulsive circling had completely disappeared. The dog did not retreat anymore, and his eating pattern and defecation were still normal. Despite the significant clinical improvement, the dog still slept a lot and seemed lethargic. The dog was polydipsic and polyuric, either due to the medication or central diabetes insipidus, which decreased after discontinuation of the phenobarbital.

Beginning at 41 days after ^166^HoMS treatment, the owners noticed a return of neurological signs, including the compulsive circling, increased anxiety, and inappropriate urination and defecation. Additionally, the dog did not understand routine commands anymore. Diagnostic imaging was repeated 51 days after treatment. CT revealed a decrease in height and width of the tumor, with still dilated lateral ventricles. MRI confirmed these findings and showed no intracranial hemorrhage, edema, or indication for local tumor regrowth. Treatment with prednisone was started (1 mg/kg, twice daily) which calmed the dog and significantly decreased the compulsive circling and behavioral changes. After 10 days, the prednisone intake was steadily decreased.

At 138 days after ^166^HoMS treatment, neurological examination showed that the dog was alert, but slow and absent-minded at times. The compulsive circling and anxiety had returned upon decreasing the prednisone. The polyuria and polydipsia had returned, and the dog preferred to lay on cold surfaces. The dilation of the ventricles and intratumoral mineralization seemed unchanged on CT, and there was still no indication for hemorrhage, edema, or local tumor regrowth. MRI was not repeated.

Because the clinical signs worsened, transsphenoidal hypophysectomy including biopsy was performed of the residual tumor five months after ^166^HoMS treatment, initially leading to clinical improvement. The neurological signs worsened rapidly two weeks after hypophysectomy and the dog was euthanized at request of the owners, by their own veterinarian and without a postmortem examination.

## Outcomes

### Tumor Response

To assess tumor response while correcting for the size of the dog, we calculated the pituitary-to-brain (P/B) ratio (cm^−1^, Equation 3), the pituitary-to-brain area (P/B_A_) ratio (Equation 4), and the pituitary-to-brain volume (P/B_V_) ratio (Equation 5) on CT during treatment and follow-up (33, 34).


(3) $$\begin{array}{l} \frac{P}{B}=\frac{\text{Pituitary height }\left(cm\right)\times 10\;}{\text{ Brain area }\left(\text{c}{\text{m}}^{2}\right)} \end{array}$$

(4) $$\begin{array}{r} \frac{P}{B_{A}}=\frac{\text{Pituitary area }\left(\text{c}{\text{m}}^{2}\right)}{\text{ Brain area }\left(\text{c}{\text{m}}^{2}\right)} \end{array}$$

(5) $$\begin{array}{l} \frac{P}{B_{V}}=\frac{\text{Pituitary volume }\left(\text{c}{\text{m}}^{3}\right)}{\text{ Brain volume }\left(\text{c}{\text{m}}^{3}\right)} \end{array}$$

**Figure 2:**
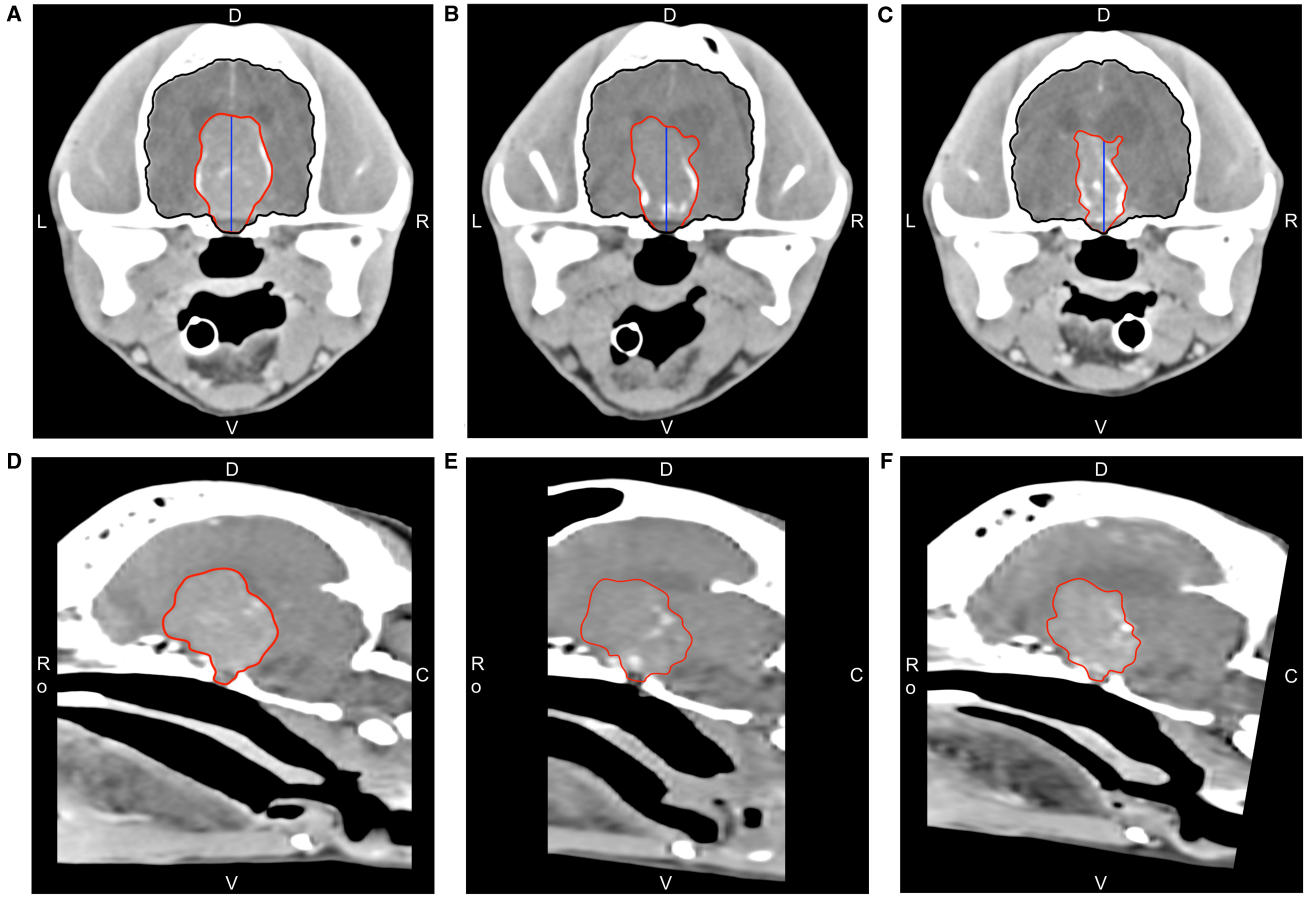
CT with delineated tumor segmentation (red), brain segmentation (black), and pituitary height (blue) used for volume and P/B ratio measurements before and after holmium-166 microsphere treatment. Coronal slices of the pituitary mass at the level of the temporomandibular joint showing the largest transverse surface area before treatment **(A)**, after 51 days **(B)**, and after 138 days **(C)**. D = Dorsal, V = Ventral, L = Left, R = Right, Ro = Rostral, C = Caudal. Corresponding mid-sagittal slices before treatment **(D)**, after 51 days **(E)**, and after 138 days **(F)**.

The P/B ratios and tumor volumes were 1.86 cm^−1^ and 8.8 cm^3^ at time of treatment, 1.69 cm^−1^ and 6.1 cm^3^ after 51 days, and 1.41 cm^−1^ and 5.3 cm^3^ after 138 days, respectively, revealing a maximum measured volume reduction of 40% (Figure 2; Supplementary Table 1).

### Dose Calculation

The calculated radioactivity in the used syringes and needle was 111 MBq at time of treatment, and no radioactivity was detected in the used disposables. The remaining 1097 MBq (90% of prepared radioactivity) was assumed to be administered into the tumor, leading to a calculated average absorbed tumor dose of 1866 Gy in 8.8 cm^3^ according to Equation 2 (Supplementary Table 1).

### Pathologic Findings

The formalin-fixed tissue samples from ^166^HoMS treatment and debulking surgery were paraffin-embedded and stained with hematoxylin and eosin (HE). Histological analysis of the samples acquired before ^166^HoMS administration contained sheets of a monotonous though moderately pleomorphic cell population of rounded to polygonal cells with amphophilic cytoplasm and variably sized round nuclei with an incidental mitotic figure. We also noted scattered areas with mineralization (probably dystrophic calcification) and multiple follicular structures filled with proteinaceous material. These findings indicated an adenoma or low-grade adenocarcinoma.

**Figure 3:**
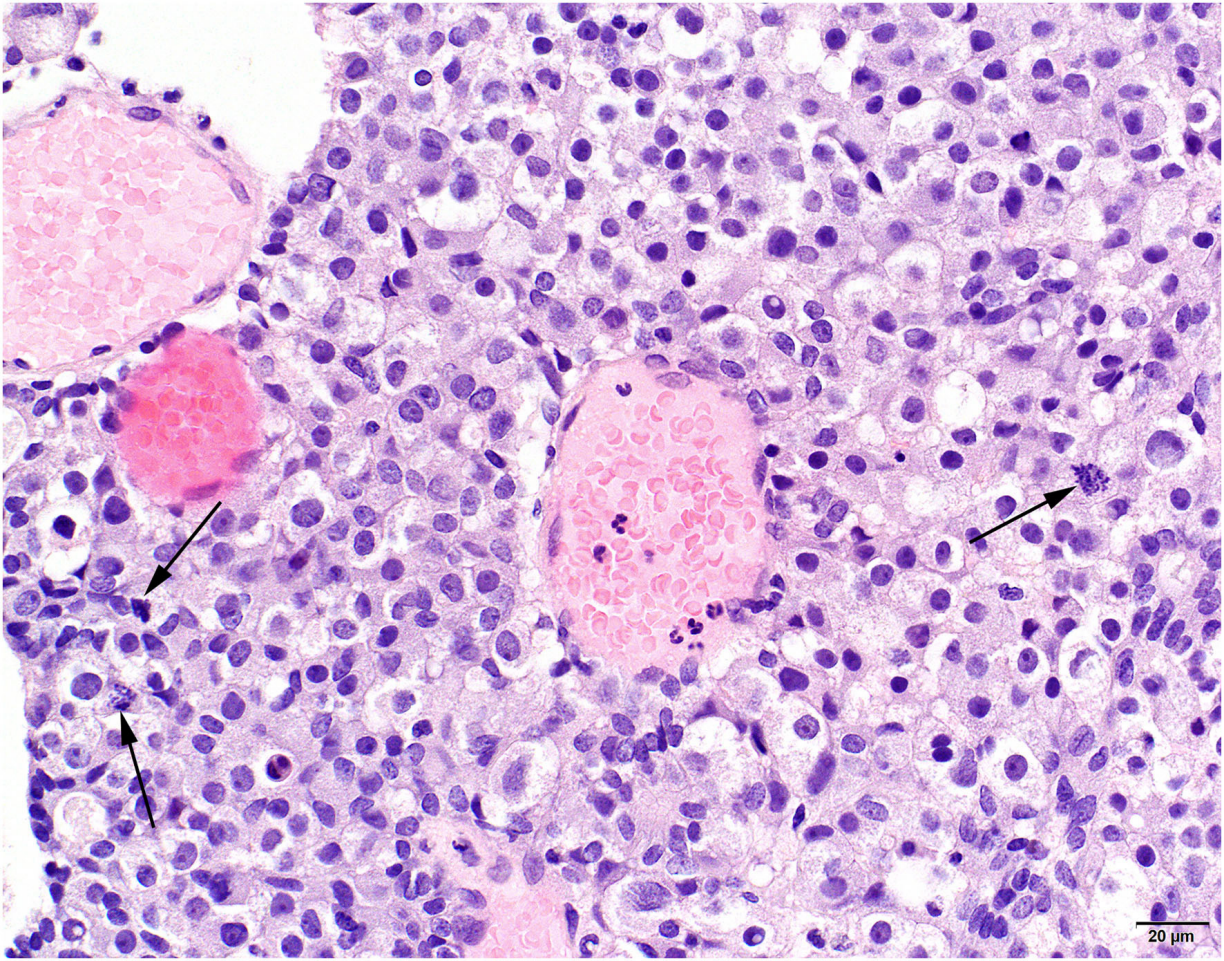
Histological view of surgical specimen of the pituitary tumor tissue 5 months after holmium-166 microsphere treatment. Basophilic neoplastic cells in sheets with pronounced variation in nuclear size and several mitotic figures (arrows). HE stains, obj. 40x.

The tissue samples acquired during debulking surgery contained a neoplastic cell population mainly consisting of basophilic cells with more prominent pleiomorphic characteristics and nuclear size variations compared to the pretreatment samples, indicating a basophilic adenocarcinoma (Figure 3). Immunohistochemical analysis showed that all tissue samples were negative for adrenocorticotropic hormone and growth hormone and had very faint non-specific staining for alpha-Melanocyte-stimulating hormone, which is consistent with a nonfunctioning (endocrine-inactive) pituitary neoplasm (35). No HoMS were found in HE-stained and Periodic acid-Schiff-stained tissue samples.

## Discussion

To our knowledge, this is a first case report describing the intracranial intratumoral treatment of a canine patient using radioactive microspheres. Despite the 40% tumor volume reduction over 138 days after ^166^HoMS treatment, the clinical signs worsened, ultimately leading to euthanasia. The remaining tumor volume at 138 days after treatment was still relatively large, 5.3 cm^3^ with a P/B ratio of 1.41 cm^−1^. Dogs with enlarged pituitaries (P/B ratio > 0.31 cm^−1^) have markedly lower survival and disease-free fractions after hypophysectomy compared to dogs with nonenlarged pituitaries (P/B ratio ≤ 0.31 cm^−1^) (36). RT can result in severe adverse effects such as pituitary hemorrhage, despite a significant decrease in P/B ratio, as observed in dogs with pituitary-dependent hyperadrenocorticism (37). RT also carries the risk of radiation necrosis of healthy brain tissue, potentially leading to death after five-to-eight months (38). In our patient, intracranial edema, hemorrhage, or local tumor regrowth were not observed on imaging at 51 and 138 days after ^166^HoMS treatment. It is unlikely that radiation necrosis of healthy brain tissue occurred in our patient as both injections were performed with the needle tip in the center of this relatively large tumor and the maximum ^166^HoMS beta radiation penetration depth is only 8.7 mm. We would not expect the HoMS suspension to cause severe adverse effects in time, as its safety and toxicity for intratumoral injection has been demonstrated (15, 16, 18, 25). Moreover, poloxamer 188 is approved by the Food and Drug Administration for IM and IV injection and has neuroprotective properties (39–41). Unfortunately, we cannot rule out adverse effects occurring later than follow-up imaging as cause for worsening of clinical signs. We did not have sufficient follow-up of our patient to assess potential long-term side-effects, and we did not have the possibility of postmortem examination and/or imaging.

The doses used for intratumoral treatment of oral SCC in cats (median 1216 Gy), in head and neck SCC in humans (median 224 Gy), and in this study (1866 Gy) are much higher than doses applied with stereotactic and external beam RT (15, 16). Pituitary tumors are often treated with fractionated RT using daily-dose fractions of 1.8-3 Gy, accounting for 45-54 Gy in total, or with stereotactic radiosurgery using one to three fractions of 10-25 Gy (10, 42–45). In comparison, the aforementioned intratumoral ^166^HoMS treatment of 13 cats with oral SCC led to a mean tumor volume decrease of 83% ± 22% in six responders (55%) with minimal side effects (15). In our patient, the injected amount of radioactivity (1097 MBq) was even higher than planned (566 MBq), because the “loss” of ^166^HoMS was lower than the anticipated 50% based on previous applications (15). In addition, the tumor volume as calculated before treatment using the ellipsoid formula (10.6 cm^3^, Equation 1) was larger compared to the segmented tumor volume on CT (8.8 cm^3^), resulting in a higher calculated average dose. Because of the centrally located injections and the limited beta radiation penetration depth, we considered it safe to inject the total amount of ^166^HoMS. The measured tumor volume reduction may be a direct result of the high local dose. On the other hand, it is likely that we did not achieve complete tumor dose-coverage with this approach as the spatial ^166^HoMS distribution inside the tumor may be more important than the absorbed dose (15, 16). To overcome this limitation, further research should focus on improving intratumoral ^166^HoMS administration by using multiple image-guided injections with intraprocedural quantitative imaging-based dosimetric feedback, and varying ^166^HoMS radioactivity adjusted to tumor size for each patient. To increase our knowledge on administration of relatively high intratumoral doses (200 Gy and higher), the resulting tumor response should also be studied, ultimately to enable complete tumor dose-coverage with higher treatment safety and efficacy.

An invasive pituitary adenoma was initially diagnosed in our patient based on clinical signs and imaging, i.e., volume of the mass, bony lysis, and the absence of intracranial metastases. However, we did not search for extracranial metastases. Our histopathology results indicated an adenoma or low-grade adenocarcinoma at time of ^166^HoMS treatment, and a nonfunctioning adenocarcinoma at time of debulking surgery. The present residual tumor cells five months after ^166^HoMS treatment are most likely a result from the incomplete tumor dose-coverage. There was no morphological indication for the presence of ^166^HoMS in the available tissue samples, possibly because (1) the acquired samples originated from the ventral tumor part, whereas the needle tip was in the tumor center during the injections, (2) the changes in tumor volume after treatment could have affected the ^166^HoMS distribution, and (3) the ^166^HoMS could have degraded or migrated after treatment as the *in vivo* stability after administration has only been tested for up to two months (18, 22, 25). SPECT could have been of added value to rule out ^166^HoMS shunting to healthy tissue elsewhere in the body.

The strength of our study is the CT-guided treatment as opposed to earlier described freehand injections (15, 16). Holmium is paramagnetic and has a high electron density, while ^166^Ho also emits gamma-rays (E_β, *max*_ = 0.081 MeV, 6.6%), enabling MRI, CT, and SPECT, respectively (46, 47). These imaging modalities have already been used successfully for ^166^HoMS quantification, biodistribution assessment, and dose calculations in radioembolization of the liver (46, 48, 49). ^166^HoMS quantification on CT after intratumoral administration was recently demonstrated with excellent correlation between the dose calibrator, SPECT, and CT in *ex vivo* tissue (50). As tumor dose-coverage was likely to be incomplete in our patient, the implementation of real-time imaging-based dosimetry is required for controlled and complete tumor dose-coverage during intratumoral ^166^HoMS treatment, to increase treatment safety and efficacy in the future.

## Conclusion

Intratumoral administration of ^166^HoMS was a feasible treatment option for an invasive pituitary adenoma in a Jack Russell Terrier, resulting in 40% tumor volume reduction in a relatively short period of time (138 days) without evident severe periprocedural side effects. Implementation of different administration techniques and imaging-based dosimetry is required for higher treatment safety and efficacy. Most importantly, prospective patient trials are required to assess the safety and survival after ^166^Ho microbrachytherapy and to compare these with conventional treatments for intracranial tumors.

## Data Availability Statement

The original contributions presented in the study are included in the article/Supplementary Material, further inquiries can be directed to the corresponding author.

## Ethics Statement

The clinical study was reviewed and approved by the Ethical Committee of the Faculty of Veterinary Medicine, Utrecht University, Utrecht, The Netherlands (protocol 2496KGD-holmium-tumoren). Written informed consent was obtained from the owners for the participation of their animals in this study.

## Author Contributions

NM and SN drafted, reviewed, and edited the manuscript. NK, BM, JK, GG, IS, JH, and JN reviewed and edited the manuscript. BM, JK, JN, and SN performed the treatment. GG performed histopathological analysis. IS and NM performed image analysis. All authors contributed to the article and approved the submitted manuscript.

## Funding

This work is part of a large research project for the development of image-guided intratumoral microbrachytherapy of brain tumors using holmium-166 microspheres. The authors declare that this study received funding from the Dutch Research Council (Grant Number 15499). The funder was not involved in the study design, collection, analysis, interpretation of data, the writing of this article or the decision to submit it for publication.

## Conflict of Interest

JN reports grants from Dutch Research Council (NWO), during the conduct of the study; personal fees from Quirem Medical, outside the submitted work also has a patent (patent family: US9731037B2, EP02077626) with royalties paid to application filed by University Medical Center Utrecht, Utrecht University Holding BV (licensed to spin-off company Quirem medical), a patent (patent family: US8691280B2, EP2178817B1, EP2017253A1) with royalties paid to Stichting voor de Technische Wetenschappen (STW), UMC Utrecht Holding BV (licensed to spin-off company Quirem medical BV), a patent (patent family: US9999695B2) with royalties paid to UMC Utrecht Holdings BV, UMC Utrecht Holding BV, a patent (Patent family: US20190368046A1) pending to BASF Corp., and a patent (patent family: WO2020122729A1) pending to Quirem Medical BV. The patents of which the patent holder is UMC Utrecht are subject to an inventor's fee. This is standard for inventors of patents working in an Academic Hospital in the Netherlands. JN is co-founder and parttime scientific advisor of Quirem Medical (Terumo) and his activities within Quirem Medical are approved and supported by the Board of Directors of the Radboudumc. The remaining authors declare that the research was conducted in the absence of any commercial or financial relationships that could be construed as a potential conflict of interest.

## Publisher's Note

All claims expressed in this article are solely those of the authors and do not necessarily represent those of their affiliated organizations, or those of the publisher, the editors and the reviewers. Any product that may be evaluated in this article, or claim that may be made by its manufacturer, is not guaranteed or endorsed by the publisher.
