# Supplementary material for: Case Report: Radioactive Holmium-166 Microspheres for the Intratumoral Treatment of a Canine Pituitary Tumor
Source: Front Vet Sci. 2021 Nov 4;8:748247. doi: 10.3389/fvets.2021.748247 (PMC8600255; doi:10.3389/fvets.2021.748247)
Supplement: Supplementary file 1 [file Table_1.docx]

**Supplementary Table 1.** Segmented brain and tumor measurements on CT at time of holmium-166 microsphere treatment (0 days), after 51 days, and after 138 days. The pituitary height (H), pituitary area (A) and brain area were measured in the transverse slice showing the largest transverse tumor area using Horos (<https://horosproject.org>). Volumes (V) were calculated by manually segmenting the brain and the tumor using Slicer (<http://www.slicer.org>). As the brain was not fully scanned during follow-up, the calculations were normalized by also using the brain volume from before treatment for the follow-up calculations. The pituitary height (cm) x 10-to-brain area (cm^2^) (P/B) ratio, the pituitary area (cm^2^)-to-brain area (cm^2^) (P/B_A_) ratio, and the pituitary volume (cm^3^)-to-brain volume (cm^3^) (P/B_V_) ratio were calculated. Maximum measured tumor volume reduction was 40% after 138 days, from 8.8 cm^3^ to 5.3 cm^3^ with a decrease in P/B ratio from 1.86 cm^-1^ to 1.41 cm^-1^.

|  | **Pituitary** | | | **Brain** | | **P/B**  (cm^-1^) | **P/B_A_** | **P/B_V_** | **Change** |
| --- | --- | --- | --- | --- | --- | --- | --- | --- | --- |
|  | H (cm) | A (cm^2^) | V (cm^3^) | A (cm^2^) | V (cm^3^) |  |  |  |  |
| 0 days | 3.10 | 4.73 | 8.84 | 16.68 | 79.58 | 1.86 | 0.28 | 0.11 | 0% |
| 51 days | 2.78 | 4.00 | 6.08 | 16.42 | 79.58 | 1.69 | 0.24 | 0.08 | -31% |
| 138 days | 2.47 | 2.65 | 5.31 | 17.52 | 79.58 | 1.41 | 0.15 | 0.07 | -40% |
